# Effectiveness of dry needling for improving pain and disability in adults with tension-type, cervicogenic, or migraine headaches: protocol for a systematic review

**DOI:** 10.1186/s12998-019-0266-7

**Published:** 2019-09-26

**Authors:** Mohammadreza Pourahmadi, Mohammad Ali Mohseni-Bandpei, Abbasali Keshtkar, Bart W. Koes, César Fernández-de-Las-Peñas, Jan Dommerholt, Mehrdad Bahramian

**Affiliations:** 1 Pediatric Neurorehabilitation Research Center, University of Social Welfare and Rehabilitation Sciences, Tehran, Iran; 2 Department of Physiotherapy, School of Rehabilitation Sciences, Iran University of Medical Sciences, Tehran, Iran; 3 University Institute of Physical Therapy, Faculty of Allied Health Sciences, University of Lahore, Lahore, Pakistan; 4 Department of Health Sciences Education Development, School of Public Health, Tehran University of Medical Sciences, Tehran, Iran; 5 Department of General Practice, Erasmus MC, University Medical Center, Rotterdam, The Netherlands; 6 Center for Muscle and Joint Health, University of Southern Denmark, Odense, Denmark; 7 Department of Physical Therapy, Occupational Therapy, Rehabilitation and Physical Medicine, Universidad Rey Juan Carlos, Alcorcón, Madrid, Spain; 8 Cátedra de Investigación y Docencia en Fisioterapia: Terapia Manual y Punción Seca, Universidad Rey Juan Carlos, Alcorcón, Madrid, Spain; 9 Bethesda Physiocare, Inc., Bethesda, MD USA; 10 Myopain Seminars, LLC, Bethesda, MD USA; 11 PhysioFitness, LLC, Rockville, MD USA; 12 Department of Physical Therapy and Rehabilitation Science, School of Medicine, University of Maryland, Baltimore, MD USA

**Keywords:** Dry needling, Cervicogenic headache, Tension-type headache, Migraine, Systematic review, Meta-analysis

## Abstract

**Background:**

Headache is the most common neurological symptoms worldwide, as over 90% of people have noted at least one headache during their lifetime. Tension-type headaches, cervicogenic headaches, and migraines are common types of headache which can have a significant impact on social, physical, and occupational functioning. Therapeutic management of headaches mainly includes physical therapy and pharmacological interventions. Dry needling is a relatively new therapeutic approach that uses a thin filiform needle without injectate to penetrate the skin and stimulate underlying tissues for the management of neuromusculoskeletal pain and movement impairments.

The main objective of this systematic review and meta-analysis is to evaluate the effectiveness of dry needling in comparison to other interventions on pain and disability in patients with tension-type headache, cervicogenic headache, and migraine.

**Methods/design:**

We will focus on clinical trials with concurrent control group(s) and comparative observational studies assessing the effect of dry needling in patients with tension-type headache, cervicogenic headache, and migraine. Electronic databases from relevant fields of research (PubMed/ Medline, Scopus, Embase®, PEDro, Web of Science, Ovid, AMED, CENTRAL, and Google Scholar) will be searched from inception to June 2019 using defined search terms. No restrictions for language of publication or geographic location will be applied. Moreover, grey literature, citation tracking, and reference lists scanning of the selected studies will be searched manually. Primary outcomes of this study are pain intensity and disability, and secondary outcomes are cervical spine ROM, frequency of headaches, health-related quality of life, and TrPs tenderness. Studies will be selected by three independent reviewers based on prespecified eligibility criteria. Three reviewers will independently extract data in each eligible study using a pre-piloted Microsoft Excel data extraction form. The assessment of risk of bias will be implemented using the Cochrane Back and Neck Review Group 13-item criteria and NOS. Direct meta-analysis will be performed using a fixed or random effects model to estimate effect size such as standardized mean difference (Morris’s *d*_*ppc*_) and 95% confidence intervals. Statistical heterogeneity will also be evaluated using the *I*^*2*^ statistic and the χ^2^ test. All meta-analyses will be performed using Stata V.11 and V.14 softwares. The overall quality of the evidence for the primary outcomes will be assessed using GRADE.

**Discussion:**

All analyses in this study will be based on the previous published papers. Therefore, ethical approval and patient consent are not required. The findings of this study will provide important information on the value of dry needling for the management of tension-type headache, cervicogenic headache, and migraine.

**Trial registration:**

PROSPERO registration number: CRD42019124125.

**Electronic supplementary material:**

The online version of this article (10.1186/s12998-019-0266-7) contains supplementary material, which is available to authorized users.

## Background

Headache is a major health concern as one of the most common type of all symptoms in the worldwide population [1, 2]. According to the 2016 Global Burden Disease study [3], “tension-type headache” and “migraine” which are described as primary headache syndromes have the third and sixth highest prevalence among 328 diseases and injuries in 195 countries from 1990 to 2016. Haldeman and Dagenais [4] reported that the prevalence of tension-type headaches, migraines, chronic daily headaches, and cervicogenic headaches in the general population is 38, 10, 3, and 0.4% to 2.5%.

Tension-type headache is identified by a bilateral pressing or tightening quality (non-pulsating quality), a mild to moderate intensity, and pain, which is not aggravated by routine physical activity, in the absence of vomiting, nausea, but may be accompanied by either photophobia or phonophobia [5–8]. These symptoms, however, do not present simultaneously during the same episode [9]. This neurological disorder is more common among female patients (female-to-man ratio of 5:4). The peak prevalence occurs between the ages of 30 and 39 [10]. The International Headache Society [5] classifies tension-type headache into three subtypes according to headache frequency: infrequent episodic (< 1 day of headache per month), frequent episodic (1–14 days of headache per month), and chronic (≥15 days per month).

Despite extensive neurophysiological and clinical studies, the exact cause of tension-type headache remains unknown [6, 7], however, peripheral nociceptive mechanisms appear to be the main cause of episodic tension-type headache, while chronic tension-type headache may be caused by central sensitization, inadequate endogenous pain control, and peripheral myofascial mechanisms (myofascial nociception) [11–15]. Previous experimental studies demonstrated that referred pain originating in myofascial TrPs within neck and shoulder muscles and surrounding soft tissues, such as fascia, tendons, and ligaments may reproduce headaches in patients with tension-type headache [16–20]. TrPs can be defined as hyperirritable palpable spots of taut fibers located within a myofascial tissue, which have been known to cause non-dermatomal referral pain and discomfort [21]. Muscles which are commonly involved in tension-type headache include the sub-occipital, sternocleidomastoid, upper trapezius, levator scapula, splenius, temporalis, and masseter [14, 22, 23].

Cervicogenic headache is characterized by chronic pain that originates from bony structures or soft tissues of the neck and is referred to the head [24]. The pain of cervicogenic headache is usually unilateral with occipitofrontal distribution of spread [25]. The prevalence of cervicogenic headache has been estimated at 15–20% in patients with chronic headaches [26]. The most accepted mechanism of cervicogenic headache is convergence between the trigeminal nerve and C_1_–_3_ nerves in the trigeminocervical nucleus [27]. The characteristics of tension-type headache and cervicogenic headache are similar, however, according to the Cervicogenic Headache International Study Group criteria [28] most cervicogenic headaches can be differentiated from tension-type headache and migraine with some overlap. In addition, according to Linde et al. [29], some patients may suffer from both types of headaches.

Migraine is defined as severe throbbing headache with nausea or vomiting associated with photophobia, that is aggravated by routine physical activity such as walking or climbing stairs [30, 31]. Migraine typically lasts between 4 and 72 h and has unilateral location [31]. Despite many migraine publications, the mechanism of migraine is not yet well understood [32]. The mechanism of migraine is believed to involve the trigeminal cervicogenic complex, which receives nociceptive information via afferent projections from the dura matter in large intracranial vessels [33]. A study conducted by Florencio et al. [34] indicated that patients with migraine exhibited active TrPs in their neck extensor muscles. According to the IHS, migraine is diagnosed if a person has at least 5 attacks fulfilling the abovementioned criteria [35].

Therapeutic management of headaches mainly comprises physical therapy and pharmacological approaches [36–38]. In the last decade, there has been an increasing interest in the use of dry needling for the treatment of headache as well as for neck and shoulder pain syndromes [38]. Dry needling is a skilled intervention frequently performed by physical therapists, physicians, chiropractors, and acupuncturists for the relief of myofascial pain disorders [39, 40]. In this technique a fine sterile needle is utilized to penetrate the skin, subcutaneous tissues, fascia, and muscle, with the goal of deactivating TrPs without the use of an anesthetic [41]. Once a TrP is deactivated, the fine needle is removed [42]. It is an efficient, easy-to-learn-and-perform procedure with a low risk profile [43]. Hong [44] suggested that local twitch responses should be elicited during dry needling for a successful technique; however, recent studies have questioned this notion [45, 46]. The time of application will rely upon the irritability of the TrP [38]. Although dry needling might not change all central sensitization aspects, it is probable that local and referred pain will be reduced, muscle blood flow, oxygenation, patterns of muscle activation, and range of motion will be improved, and the biochemical environment of TrPs will be changed [47–49]. Linde et al. [50] reported that the physiological mechanism of dry needling includes a combination of peripheral effects (such as spinal [i.e., gate control] and supraspinal [i.e., endogenous opioid system] mechanisms), as well as cortical effects (such as psychological or placebo mechanisms). It is hypothesized that dry needling may activate the serotonergic (5-HT) and noradrenergic descending inhibitory systems, which in turn may decrease pain [48]. Furthermore, Cagnie et al. [48] hypothesized that dry needling, via stimulation of the nociceptive fibers, may stimulate the enkephalinergic inhibitory dorsal horn interneurons. It is unclear whether the needle manipulation or the electrical stimulation is responsible for these results or both [48].

To the best of our knowledge, a systematic review conducted by France et al. [6] has investigated the effectiveness of dry needling and conventional physiotherapy in the management of cervicogenic headache or tension-type headache. Ten electronic databases were searched up to October 2012 and three relevant studies (two clinical trials and one case report) were identified through searches. Two included clinical trials with tension-type headache participants (40 male and 35 female) demonstrated statistically significant improvements following dry needling, but no significant differences between groups [6]. Furthermore, one case report study with a cervicogenic headache female that was included in the systematic review showed significant improvement in pain and neck disability index after nine treatment sessions of dry needling combined with manual therapy [6]. A formal meta-analysis was not performed because the number of studies included was not enough [6]. Additionally, grey literature was not included in the former systematic review to assure the comprehensiveness of the search strategy [6]. Although no systematic review and meta-analysis studies have been conducted to evaluate the effectiveness of dry needling on headache, several Cochrane systematic review studies have looked at the effectiveness of acupuncture in headaches [29, 50, 51]. In 2016, Linde et al., [29] investigated whether acupuncture is more effective than routine care, than ‘sham’ acupuncture; and other interventions in reducing headache frequency in adults with episodic or chronic tension-type headache. Twelve randomized trials with 2349 participants (median 56, range 10 to 1265) were included in the present systematic review and the results indicated that acupuncture is effective for treating frequent episodic or chronic tension-type headaches, but further trials - particularly comparing acupuncture with other treatment options such as physical therapy, massage or exercise - are needed [29]. In another systematic review, Linde et al. [51] assessed the effectiveness of acupuncture in reducing headache frequency in patients with migraine. Twenty-two trials with 4419 participants (median 42, range 27 to 1715) were included and the results showed that there was consistent evidence that acupuncture provides additional benefit to treatment of acute migraine attacks only or to routine care. However, Linde et al. [51] found no evidence for an effect of ‘true’ acupuncture over ‘sham’/‘placebo’ acupuncture. Moreover, it has been suggested that acupuncture is at least as effective as, or possibly more effective than, prophylactic drug treatment, and has fewer adverse effects [51]. Finally, Linde et al. [51] concluded that acupuncture should be considered a treatment option for patients with migraine willing to undergo this treatment.

Despite the widespread use of dry needling in the treatment of headaches, its effectiveness is still controversial when compared with other techniques. Furthermore, because the previous published systematic review on this topic is out of date, a new systematic review of the literature is needed. Hence, the main objective of this systematic review and meta-analysis is to evaluate the effectiveness of dry needling in comparison to other interventions on pain and disability in patients with tension-type headache, cervicogenic headache, and migraine.

## Methods

This systematic review will be performed in accordance with the PRISMA statement [52] and principles outlined in the *Cochrane Handbook for Systematic Reviews of Interventions* [53]. This protocol has been prepared with regard to the PRISMA-P 2015 guidelines [54] and was registered on PROSPERO (International Prospective Register of Systematic Reviews, http://www.crd.york.ac.uk/PROSPERO/; #CRD42019124125) in 4 March 2019. Ethical approval and patient consent will not be required since this is a systematic review of previously published studies and no new data collection will be undertaken.

### Search strategy and study selection

A comprehensive electronic database search will be performed from inception to June 31, 2019 on the following databases: Medline (NLM) via the PubMed, Scopus, Embase®, PEDro, Web of Science, Ovid, AMED via the EBSCO, CENTRAL via The Cochrane Library, and Google Scholar. Electronic search strategies are constructed based on the combined keywords: *tension-type headache, cervicogenic headache, migraine,* and *dry needling* to identify human studies in the literature that investigated the effectiveness of dry needling in adult patients (≥ 18 years) with tension-type headache, cervicogenic headache, or migraine. A combination of MeSH (Medline), Emtree (Embase®) terms, and free text words in research equations with ‘OR’ and ‘AND’ Boolean operators will be used. Free text words will be selected from the indexed keywords of most relevant original studies and reviews in Scopus. To retrieve all possible variations of a specific root word, wildcards, and truncations will also be applied. The search strategy is customized according to the database being searched. In addition, if additional keywords of relevance are detected during electronic searches we will modify and re-formulate the search strategies to incorporate these terms. Three authors (M.R.P., M.A.M.B., and M.B.) will develop the sufficient search syntax, and after piloting and finalizing it, the search of the electronic databases will be conducted by one author (M.R.P.). Moreover, we will consult a biomedical librarian to review our search strategy using the PRESS 2015 guideline evidence-based checklist [55] in order to minimize error in our search strategies. Details of PubMed/Medline (NLM) database search syntax are presented in Additional file 1. PubMed’s ‘My NCBI’ (National Center for Biotechnology Information) email alert service will be employed for identification of newly published systematic reviews using a basic search strategy.

Citation tracking and reference lists scanning of the selected studies and relevant systematic reviews will be searched for eligible studies. Manual search of keywords via internet will be also conducted. Additionally, the table of contents of the journal of *Cephalalgia* and the *Journal of Bodywork & Movement Therapies* will be reviewed. The key journals are identified within the research in the Web of Science and Scopus. To minimize publication bias, grey literature will be identified by searching for conference proceedings (via ProQuest, Scopus, and Web of Science Conference Proceedings Citation Index database), unpublished masters and doctoral theses (via ProQuest and OpenGrey; System for Information on Grey Literature in Europe), and unpublished trials (via US National Institutes of Health Ongoing Trials Register [ClinicalTrials.gov], WHO International Clinical Trials Registry Platform, and International Standard Randomized Controlled Trials Number [ISRCTN]. Abstracts from the annual meeting of American Headache Society and European Headache Federation congress in the last 5 years and abstracts from the congress of the International Headache Society in the last 4 years will also be searched. In addition, experts with clinical and research experience on the role of dry needling for headaches will be consulted. Finally, one author (M.R.P.) will complete the search process by manual searching in Google. We will not review content from file sources that are from mainstream publishers (e.g., BMJ, Sage, Wiley, ScienceDirect, Springer, and Taylor & Francis), as we expect these to be captured in our broader search strategy.

If a full text of a relevant article is not accessible, a contact will be made with the corresponding author(s). In addition, when unpublished works are retrieved in our search, an email will be sent to the corresponding author(s) to determine whether the work has been subsequently published. If no response received from the corresponding author(s) after three emails, the study will be excluded.

### Eligibility criteria

All publications identified by the searches will be imported into the EndNote reference management software (version X9.1; Clarivate Analytics Inc., Philadelphia, PA, USA), and duplicates will be removed automatically and double-checked manually. The titles and abstracts of each citation will be screened independently by three reviewers (M.R.P. M.A.M.B., and M.B.) according to a checklist that is developed for this purpose (Table 1) with the following criteria:

1. Study design should be clinical trials with concurrent comparison group(s) or comparative observational studies;
2. Study participants should have at least one of the three types of headache (tension-type headache, cervicogenic headache, and/or migraine);
3. Study participants should be ≥18 years of age;
4. The studies should have at least one of the primary outcomes (i.e., pain and disability) of this review; and,
5. Dry needling should be the main intervention in the study.

**Table 1:** PICOS criteria for the study

Criteria	Inclusion
Population	The population will comprise adult patients (≥ 18 years of age) of either gender with tension-type headache, cervicogenic headache, or migraine. Tension-type headache, cervicogenic headache, and migraine diagnoses must be based on the International Classification of Headache Disorders [ICHD-3 beta 2013 [35] and its previous editions ICHD-2 2004 [5]] proposed by the International Headache Society. If no specified criteria were reported among the studies, the diagnosis must be based on discriminable and important characteristics of tension-type headache, cervicogenic headache, and migraine disorders, as confirmed by patients’ doctors. Important characteristics of tension-type headache include dull, aching, non-throbbing pain occurring in short episodes of variable duration or continuously (chronic form) that can be distributed unilaterally or bilaterally and referred to temporal, parietal, occipital, or frontal regions of the head without nausea, visual/auditory disturbances, or vomiting [56]. Important characteristics of cervicogenic headache include unilateral headache with symptoms and signs of neck involvement, including impairment in cervical spine ROM and pain on palpation of the neck structures, especially on the upper cervical spine [57]. However, patients with cervicogenic headache may have headache without neck pain [35]. Finally, important characteristics of migraine attack include recurrent headache, unilateral pain, pulsating quality, moderate to severe intensity, in association with nausea and/or photophobia and phonophobia and is aggravated by physical activity [58]. Studies in which children or adolescents were treated with dry needling technique will be excluded from this systematic review.
Intervention	Dry needling is a therapeutic procedure used in subjects with myofascial pain or motor dysfunction that comprises inserting thin filiform needle directly into the skin, subcutaneous tissues, fascia, and muscle, with the intent to deactivate TrPs without the use of an anesthetic [59]. In this technique, the needle usually moves in vertical direction at approximately 1 Hz with or without rotations [38]. Sometimes the needles may be left in place for approximately 20 min with or without manual stimulation [60]. Simons et al. [61] mentioned that dry needling targeting TrPs can disrupt the dysfunctional neuromuscular activity in the muscles, decrease muscle tone, and normalize the neurochemical pathways of muscles. Because of the potential interchangeable use of terms ‘*dry needling*’ and ‘*acupuncture’* in the literature [6, 38], both search terms will be used. To be eligible for the review, an explicit explanation of the dry needling intervention is required and needed to involve the insertion of fine needles precisely into identified TrPs with the aim of influencing headache intensity or frequency [6]. Additionally, if in a study at least one local twitch response was obtained during a needling procedure, it will be considered as a dry needling technique. No restriction for dosage of dry needling, frequency of treatment sessions, duration of intervention, and time to outcome measure will be set. However, for an article to be included in this systematic review, it should have at least one session of dry needling for patients with tension-type headache, cervicogenic headache, or migraine. We will exclude acupuncture studies in which the fine needle was only inserted into acupoints on the neck, face, and head regions without obtaining at least one local twitch response. Furthermore, when dry needling is used combined with other treatment(s) in primary studies, at least 50% of the total treatment programme is required to be presented for inclusion.
Comparator	Other physical therapy/conservative interventions, different substances injections, pharmacological interventions, and sham or control group.
Outcomes	The primary outcomes of this systematic review are pain and disability. These outcomes were selected as the primary outcomes, since pain and disability are considered common patient reported outcomes. Pain is defined as pain intensity, measured at the time point closest to the end of treatment [62]. Pain intensity may be assessed with a continuous self-report scale (e.g., NPRS or VAS), a rating scale within a composite measure of pain (e.g., McGill Pain Questionnaire), or an ordinal scale with greater than six levels (we will treat such ordinal scales as continuous variables). We will not exclude studies that use other measurement tools. Functional disability is defined as any restriction or lack of ability to perform an activity in the manner or within the range considered normal for a human [63]. Functional disability may be measured with a continuous, self-report scale (e.g., FRI), a rating scale within a composite measure (e.g., social functioning scale in the Short Form-36), or an ordinal scale with greater than six levels (we will treat such ordinal scales as continuous variables). We will not exclude studies that use other measurement tools. For articles to be included, they should have at least one of the primary outcomes of this study. In addition, the secondary outcomes are cervical spine ROM, frequency of headaches, health-related quality of life (e.g., Short Form-36), and TrPs tenderness. We will exclude any other clinical or health-related outcomes from this review.
Study Design	Clinical trials with concurrent comparison group(s) as well as comparative observational studies published in peer-reviewed journals will be included in the present systematic review. Results obtained from other observational studies (i.e., cross-sectional and cohort studies without any comparison group(s)), opinion pieces, editorials, systematic reviews, narrative reviews, case reports, book chapters, policy documents, commercial documents, and websites will be excluded. Qualitative studies will not be included. Studies including tension-type headache and migraine patients with medication-overuse history according to the ICHD-3 beta criteria [35] will also be excluded. No restrictions for language of publication or geographic location will be applied. Articles published in a non-English language will be translated appropriately by Bing Microsoft Translator (https://www.bing.com/translator).

**Fig. 1:**
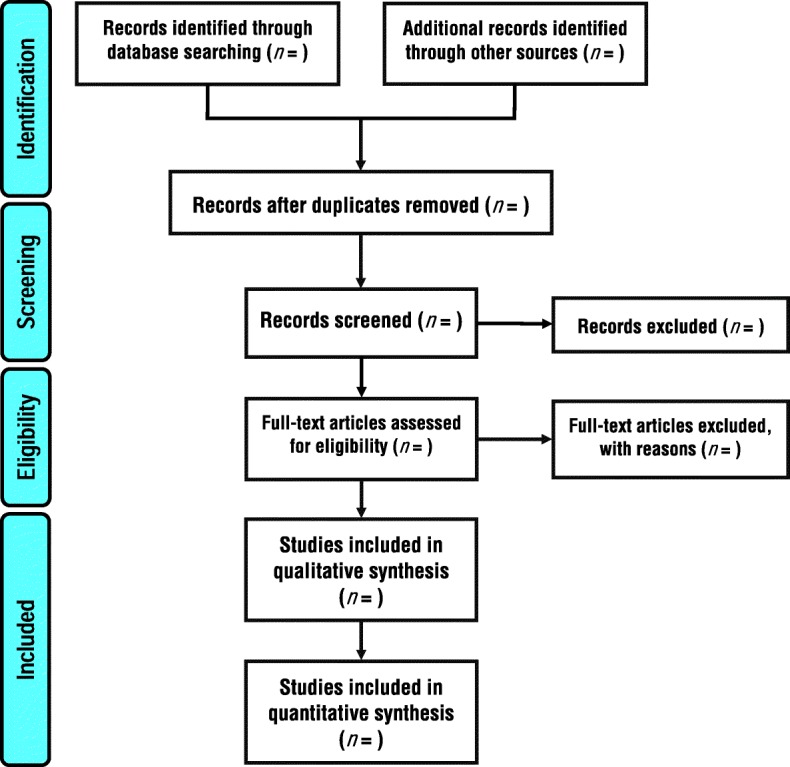
Flow diagram of study selection process

If a study meets all of the criteria, then the full-text of the study will be assessed for eligibility. In addition, a full-text review will be undertaken if the title and abstract do not provide adequate information. The selection process will be conducted strictly according to the inclusion and exclusion criteria by three independent reviewers simultaneously (M.R.P., M.A.M.B., and M.B.) (Table 1). The three reviewers are physical therapists with experience in performing systematic reviews. Disagreements will be resolved by discussion and if necessary, consultation with a fourth reviewer (A.A.K.). The eligibility criteria are based on the PICOS acronym (Table 1) and will be piloted prior to conducting the review proccess. The entire process of study selection is summarized in the PRISMA flow diagram (Fig. 1).

### Data collection and analysis

#### Risk of bias

The risk of bias of each clinical trial will be evaluated independently by three reviewers (M.R.P., M.A.M.B., and M.B.) using the Cochrane Back and Neck Review Group 13-item criteria [64]. The guideline examines six specific domains of bias, and the scoring criteria for each item in each of the domains are “Yes,” “No,” and “Unclear” if there is insufficient information to make an accurate judgment. We will categorize studies as “low risk” (at least six of the 13 criteria are met) or “high risk” (less than six criteria are met) [65]. In addition, the risk of bias assessment of each comparative observational study will be judged independently by the same reviewers (M.R.P., M.A.M.B., and M.B.) on the basis of the NOS [66]. The NOS is recommended by the Cochrane Non-Randomized Studies Methods Working Group to assess the quality of observational studies. The scale is based on the following three subscales: Selection (4 items), Comparability (1 item), and Outcome or Exposure (3 items) [67]. A total score of 3 or less will be considered high, 4–6 will be considered moderate, and ≥ 7 will be deemed low risk of bias [68]. Unacceptable bias will be defined as a zero score in any of the NOS subscales. The level of inter-rater agreement will be assessed using weighted Cohen’s kappa coefficient, with a method developed for comparing the level of agreement with categorical data along with their respective 95% confidence intervals (κ 0–0.20 = poor agreement; 0.21–0.40 = fair agreement; 0.41–0.60 = moderate agreement; 0.61–0.80 = good agreement; and 0.81–1 = very good agreement) [69]. Disagreements will be resolved by discussion and where it is required with input from a fourth reviewer (A.A.K).

The graphical presentation of assessment of risk of bias will be generated by Review Manager Software (RevMan V.5.3.5) or Stata V.14 (Sata Corp., College Station, TX, USA).

#### Data extraction

Data extraction and abstraction from each eligible study will be performed independently by three reviewers (M.R.P., M.A.M.B., and M.B.), using a Microsoft Excel spreadsheet (Microsoft, Redmond, Washington, USA) which will be designed according to the Cochrane meta-analysis guidelines and will be adjusted to the needs of this review. The data-extraction form will be pilot-tested before its use. Pilot testing will be performed on two published studies which are not included in the present systematic review but are relatively similar to the eligible studies. During pilot-testing, we will assess the characteristic of the variables (e.g., categorical or continuous) and whether all pre-defined variables in the data-extraction form are useful for the systematic review and meta-analysis. Moreover, we will check if it is possible to include additional variables in the data-extraction form in order to perform further post-hoc sensitivity analyses. The following data will be extracted from all the eligible studies:

1. *Study characteristics:* first author’s name, journal’s name, publication year, country of study performance, study year, study design, single versus multicenter, size of the sample, and duration of follow-up.
2. *Participants’ characteristics:* ethnicity, age, gender, body mass, stature, BMI, and type of headache.
3. *Intervention and comparator details:* sample size for each treatment group, muscles name, features of dry needling treatment (such as type of dry needling [superficial or deep], needle size, needling technique, and whether the technique elicited local twitch response), features of control interventions (sham/placebo methods or standard treatment details), duration of treatment session, frequency of treatment sessions per week or month, withdrawals, dropouts, and any other relevant detail.
4. *Outcome measures:* pain intensity, scales and questionnaires used to assess pain, total score of functional disability, disability questionnaires, cervical spine ROM, instruments used to measure cervical spine ROM, questionnaire used to measure health-related quality of life, and instruments used to assess TrPs tenderness. Primary and secondary outcomes will be documented at both baseline and endpoint.

Following the completion of this process, one author (M.R.P.) will double-check the extracted data to avoid any omissions or inaccuracies.

#### Dealing with missing data

If there are missing data or insufficient details in relation to the characteristics of the studies included in the meta-analysis, we will try to contact the study authors for further information. However, if the authors do not respond to queries, we will apply the following strategies to address missing data:

1. If ITT analyses were conducted in the eligible studies, we will use the ITT data instead of missing data as the first option.
2. For continuous missing outcome data, we will try to re-calculate mean difference, standard deviation, or effect size values when the test statistics, medians, *p*-values, standard errors, or confidence intervals are reported in the selected studies using the Campbell Collaboration effect size calculator (http://www.campbellcollaboration.org/escalc/html/EffectSizeCalculator-SMD-main.php).
3. If required data are presented only in graphs of the included studies, we will extract the data by using WebPlotDigitizer V.4.2 (https://automeris.io/WebPlotDigitizer/index.html).
4. If none of the above strategies can be implemented, we will try to estimate mean difference and standard deviation values from the most similar study [65, 70].

#### Assessment of heterogeneity

Statistical heterogeneity among the included studies will be assessed using the *I*^*2*^ statistic and Q test (χ^2^) as recommended by the Cochrane Handbook for Systematic Reviews of Interventions [71]. The *I*^*2*^ statistic will be interpreted using the following guide: 0–40% = no important heterogeneity; 30–60% = moderate heterogeneity; 50–90% = substantial heterogeneity; 75–100% = considerable heterogeneity [72]. Heterogeneity will be considered before conducting pooled analysis. When *I*^*2*^ values are higher than 50% and there is overlap between the confidence intervals of the included studies with the summary estimate on the forest plot, the results of all eligible studies will be combined. The potential sources of heterogeneity will be explored by sensitivity and subgroup analyses/meta-regression.

#### Assessment of publication bias

Publication bias will be explored by constructing funnel plot and performing Begg and Mazumdar’s rank correlation [73] and Egger’s linear regression tests [74]. A *p*-value < 0.05 for Begg and Mazumdar’s rank correlation and Egger’s linear regression tests indicates significant statistical publication bias. However, the *p*-value will be set at 0.10 if the number of included studies is < 10. Moreover, Duval and Tweedie ‘trim and fill’ method will be conducted to explore the potential influence of a publication bias [72]. Publication bias will not be assessed by constructing funnel plot when < 10 studies are available per primary outcome of interest, since the plot for publication bias yields unreliable results [70]. Publication bias will be assessed using Stata V.14 (Stata Corp., College Station, TX, USA).

### Data synthesis

#### Statistical analysis

Pooled effects of continuous variables will be expressed as Morris’s delta (Morris’s *d*_*ppc*_), if the same primary outcomes are used in the eligible studies. Morris described a pre-post control effect size as “*the mean pre-post change in the treatment group minus the mean pre-post change in the control group, divided by the pooled baseline standard deviation of both the treatment and control groups*” [75, 76]:

$$ {d}_{ppc}={c}_p\left[\frac{\left({M}_{post,T}-{M}_{pre,T}\right)-\left({M}_{post,C}-{M}_{pre,C}\right)}{SD_{pre}}\right] $$

The pooled pretest standard deviation is calculated as [75, 76]:

$$ {SD}_{pre}=\sqrt{\frac{\left({n}_T-1\right){SD^2}_{pre,T}+\left({n}_C-1\right){SD^2}_{pre,C}}{n_T+{n}_C-2}} $$ *T*: treatment; *C*: control

The small sample size bias-correction is calculated as [75, 76]:

$$ {C}_P=1-\frac{3}{4\left({n}_T+{n}_C-2\right)-1} $$

Effect size (Morris’s *d*_*ppc*_) will be calculated using Campbell Collaboration effect size calculator (http://www.campbellcollaboration.org/escalc/html/EffectSizeCalculator-SMD-main.php) and Psychometrica online tool (https://www.psychometrica.de/effect_size.html#cohc). If continuous outcomes measures are different between studies, we will also express pooled effects with Morris’s *d*_*ppc*_, but we will first convert the different outcome measures to a 0 to 100 scale [65]. For the measurement of effect sizes three levels are defined: small effect size (*d*_*ppc*_ < 0.40), medium effect size (0.40 ≤ *d*_*ppc*_ ≤ 0.70) or large effect size (*d*_*ppc*_ > 0.70). Although there are no available data for minimally clinically important differences (MCIDs) for pain and disability in adult patients with headache, a clinically important effect for the primary outcomes is considered when the magnitude of the effect size is at least medium [65]. Meta-analysis will be done separately on studies with clinical trial design and on studies with comparative observational design. Additionally, meta-analyses will be conducted separately on tension-type headache, cervicogenic headache, and migraine within each study design. In the presence of a sufficient number of studies, we will also conduct a priori subgroup analysis based on the overall risk of bias score (high, moderate, and low risk of bias). All data from the meta-analyses with 95% confidence intervals will be reported in forest plots. The random-effect model with DerSimonian–Laird (D + L) method [77] will be used to pool the data from individual studies. Stata V.11 and V.14 (Stata Corp., College Station, TX, USA) will be used for meta-analysis. Wherever applicable, NNT will be presented to help the reader better understand how the results can be applied to the individual patient. The Campbell Collaboration effect size calculator and Psychometrica online tool will be used to calculate NNT.

In addition, where a quantitative synthesis will not be deemed suitable due to low number of studies, a qualitative synthesis of results will be undertaken. We will conduct meta-analysis when ≥2 studies are available since “two” is the minimum number of studies required for meta-analysis [78]. If meta-analysis is not possible, we will summarize study results as either statistically significant (*p-*value < 0.05) or nonsignificant and calculate the effect of intervention on the outcomes of this study.

#### Unit of analysis issues

The unit of analysis will be based on aggregated outcome data as individual patient data is not available for any study.

#### Analysis problems

If sufficient homogeneous studies are available for statistical pooling, a meta-analysis will be performed for the time points: short (< 3 months after the baseline measurements were taken), intermediate (at least 3 months but < 12 months after the baseline measurements were taken) and long-term (12 months or more after the baseline measurements were taken) follow-up. If multiple time points fall within the same category, the one that is closest to the end of the treatment, 6 and 12 months will be used [70].

#### Sensitivity analysis

Sensitivity analysis using the leave-one-out method will be performed to determine the effect of each individual study on the pooled results [79]. Furthermore, sensitivity analyses will be conducted by using only high-quality studies in the meta-analyses to explore the robustness of conclusion. All sensitivity analysis will be performed using Stata V.14 (Stata Corp., College Station, TX, USA).

#### Summary of evidence

The overall quality of the evidence and strength of the recommendations for the primary outcomes will be assessed using GRADE [80]. The ‘Summary of findings’ tables will be generated by the GRADE working group online tool (GRADEpro GDT (www.gradepro.org)). The downgrading process is based on five domains: study limitations (e.g., risk of bias), inconsistency (e.g., heterogeneity between studies results), indirectness of evidence (including other patient populations or use of surrogate outcomes), imprecision (e.g., small sample size) and reporting bias (e.g., publication bias). The quality of evidence is classified as the following: (*i*) high quality—further research is unlikely to change confidence in the estimate of effect; the Cochrane criteria and NOS identify no risks of bias and all domains in the GRADE classification are fulfilled. In addition, further research is unlikely to change confidence in the estimate of effect (*ii*) moderate quality—further research is likely to have an important impact on the confidence in the estimate of effect, and one of the domains in the GRADE classification is not fulfilled; (*iii*) low quality—further research is likely to have an important impact on the confidence and may change the estimate; two of the domains are not fulfilled in the GRADE classification; and (*iv*) very low quality—we are uncertain about the estimate; three of the domains in the GRADE classification are not fulfilled [70, 80].

## Discussion

Headaches are one of the main reasons for absenteeism from works or avoid physical and social activities [81]. From 2007 to 2017, the number of all-age years lived with disabilities attributed to headaches increased by 15.4% (95% UI, 14.6–16.2) [2]. Tension-type headaches, cervicogenic headaches, and migraines are three common types of headache which can have a considerable impact on individuals’ quality of life. Physical therapy is a treatment option that consists of interventions such as manual therapy, electrotherapy, exercises, and various maneuvers in order to improve pain, disability, and quality of life in patients with headaches. Dry needling is a physical therapy modality that involves inserting a fine filiform needle into the TrPs of soft tissues. There are many theoretical models that have influenced physical therapists and clinicians practicing dry needling [82]. The ‘fast-in-and-fast-out’ technique described by Hong [44] is probably one of the most widely used for the managements of neuromusculoskeletal pain and dysfunction [82].

Despite an increasing number of studies evaluating the effectiveness of dry needling for musculoskeletal disorders, no systematic review with meta-analysis has been carried out to examine the effectiveness of dry needling in patients with headaches. It is hoped that this study will provide useful information for physical therapists and clinicians on the treatment of tension-type headache, cervicogenic headache, and migraine.

### Limitations

This review will not capture any studies that assess the secondary outcomes (i.e., cervical spine ROM, frequency of headaches, health-related quality of life, and TrPs tenderness) but did not report on pain or disability. Therefore, the findings regarding the secondary outcomes will be limited by the included studies based on the eligibility criteria.

## Additional file

Additional file 1: Search strategies for PubMed/Medline (NLM), Scopus, Web of Science, and Embase®. (DOCX 24 kb)

## Data Availability

This study is the protocol for a systematic review and materials are not being collected and no data is yet available. After publishing the systematic review results, the dataset will be available from the corresponding author on a reasonable request.

## Abbreviations

AMED: Allied and Complementary Medicine
BMI: Body mass index
CENTRAL: Cochrane Central Register of Controlled Clinical Trials
FRI: Functional Rating Index
GRADE: Grading of Recommendations Assessment, Development and Evaluation
IHS: International Headache Society
ITT: Intention to-treat
MeSH: Medical Subject Heading
NLM: National Library of Medicine
NNT: Number-needed-to-treat
NOS: Newcastle-Ottawa Scale
NPRS: Numeric Pain Rating Scale
PEDro: Physiotherapy Evidence Database
PICOS: Participants, intervention, comparison, outcomes, study design
PPC: Pre-post change
PRESS: Peer Review of Electronic Search Strategies
PRISMA: Preferred Reporting Items for Systematic Reviews and Meta-analyses
PRISMA-P: Preferred Reporting Items for Systematic review and Meta-analysis Protocols
ROM: Range of motion
TrPs: Trigger points
UI: Uncertainty interval
US: United States
V: Version
VAS: Visual analogue scale
WHO: World Health Organization
