# Supplementary material for: Effectiveness of dry needling for improving pain and disability in adults with tension-type, cervicogenic, or migraine headaches: protocol for a systematic review
Source: Chiropr Man Therap. 2019 Sep 26;27:43. doi: 10.1186/s12998-019-0266-7 (PMC6761714; doi:10.1186/s12998-019-0266-7)
Supplement: Supplementary file 1 — Search strategies for PubMed/Medline (NLM), Scopus, Web of Science, and Embase®. (DOCX 24 kb) [file 12998_2019_266_MOESM1_ESM.docx]

**Additional file 1:** Search strategies for PubMed/Medline (NLM), Scopus, Web of Science, and Embase^®^.

| **Search strategy for PubMed/Medline (NLM)** |
| --- |

(((headache*[tiab] AND tension-type[tiab]) OR (headache*[tiab] AND “tension type”[tiab]) OR (idiopathic[tiab] AND headache*[tiab]) OR (stress[tiab] AND headache*[tiab]) OR (tension[tiab] AND headache*[tiab]) OR (pressure[tiab] AND headache*[tiab]) OR “pressure headache”[tiab] OR “pressure headaches”[tiab] OR (psychogenic[tiab] AND headache*[tiab]) OR (tension-vascular[tiab] AND headache*[tiab]) OR (“tension vascular”[tiab] AND headache*[tiab]) OR “tension vascular headache”[tiab] OR “tension vascular headaches”[tiab] OR (post-traumatic[tiab] AND headache*[tiab]) OR (“post traumatic”[tiab] AND headache*[tiab]) OR (cervicogenic[tiab] AND headache*[tiab]) OR (migraine[tiab] AND disorder*[tiab]) OR migraine*[tiab] OR (migraine[tiab] AND headache*[tiab]) OR (“acute confusional”[tiab] AND migraine*[tiab]) OR “status migrainosus”[tiab] OR hemicrania[tiab] OR “status hemicranicus”[tiab] OR “familial migraine”[tiab] OR (migraine[tiab] AND disorder*[tiab]) OR (hemicrania[tiab] AND migraine*[tiab]) OR (migraine[tiab] AND variant*[tiab]) OR (sick[tiab] AND headache*[tiab]) OR (abdominal[tiab] AND migraine*[tiab]) OR “secondary headache disorders”[tiab] OR (“headache disorders”[tiab] AND secondary[tiab]) OR (“headache disorder”[tiab] AND secondary[tiab]) OR “secondary headache disorder”[tiab] OR “secondary headache”[tiab] OR “secondary headaches”[tiab] OR (cervical[tiab] OR “migraine syndromes”[tiab]) OR “chronic tension headache”) AND (“dry needle”[tiab] OR “dry needling”[tiab] OR “dry needles”[tiab] OR dry-needl*[tiab] OR needl*[tiab] OR pharmacopuncture[tiab] OR acupuncture[tiab] OR “intramuscular stimulation”[tiab] OR “intramuscle stimulation”[tiab] OR “intramuscular stimulations”[tiab] OR “intramuscle stimulations”[tiab]))

| **Search strategy for Scopus** |
| --- |

(((TITLE-ABS(headache*) AND TITLE-ABS(tension-type)) OR (TITLE-ABS(headache*) AND TITLE-ABS(“tension type”)) OR (TITLE-ABS(idiopathic) AND TITLE-ABS(headache*)) OR (TITLE-ABS(stress) AND TITLE-ABS(headache*)) OR (TITLE-ABS(tension) AND TITLE-ABS(headache*)) OR (TITLE-ABS(pressure) AND TITLE-ABS(headache*)) OR TITLE-ABS(“pressure headache”) OR TITLE-ABS(“pressure headaches”) OR (TITLE-ABS(psychogenic) AND TITLE-ABS(headache*)) OR (TITLE-ABS(tension-vascular) AND TITLE-ABS(headache*)) OR (TITLE-ABS(“tension vascular”) AND TITLE-ABS(headache*)) OR TITLE-ABS(“tension vascular headache”) OR TITLE-ABS(“tension vascular headaches”) OR (TITLE-ABS(post-traumatic) AND TITLE-ABS(headache*)) OR (TITLE-ABS(“post traumatic”) AND TITLE-ABS(headache*)) OR (TITLE-ABS(cervicogenic) AND TITLE-ABS(headache*)) OR (TITLE-ABS(migraine) AND TITLE-ABS(disorder*)) OR TITLE-ABS(migraine*) OR (TITLE-ABS(migraine) AND TITLE-ABS(headache*)) OR (TITLE-ABS(“acute confusional”) AND TITLE-ABS(migraine*)) OR TITLE-ABS(“status migrainosus”) OR TITLE-ABS(hemicrania) OR TITLE-ABS(“status hemicranicus”) OR TITLE-ABS(“familial migraine”) OR (TITLE-ABS(migraine) AND TITLE-ABS(disorder*)) OR (TITLE-ABS(hemicrania) AND TITLE-ABS(migraine*)) OR (TITLE-ABS(migraine) AND TITLE-ABS(variant*)) OR (TITLE-ABS(sick) AND TITLE-ABS(headache*)) OR (TITLE-ABS(abdominal) AND TITLE-ABS(migraine*)) OR TITLE-ABS(“secondary headache disorders”) OR (TITLE-ABS(“headache disorders”) AND TITLE-ABS(secondary)) OR (TITLE-ABS(“headache disorder”) AND TITLE-ABS(secondary)) OR TITLE-ABS(“secondary headache disorder”) OR TITLE-ABS(“secondary headache”) OR TITLE-ABS(“secondary headaches”) OR (TITLE-ABS(cervical) OR TITLE-ABS(“migraine syndromes”)) OR TITLE-ABS(“chronic tension headache”)) AND (TITLE-ABS(“dry needle”) OR TITLE-ABS(“dry needling”) OR TITLE-ABS(“dry needles”) OR TITLE-ABS(dry-needl*) OR TITLE-ABS(needl*) OR TITLE-ABS(pharmacopuncture) OR TITLE-ABS(acupuncture) OR TITLE-ABS(“intramuscular stimulation”) OR TITLE-ABS(“intramuscle stimulation”) OR TITLE-ABS(“intramuscular stimulations”) OR TITLE-ABS(“intramuscle stimulations”)))

| **Search strategy for Web of Science** |
| --- |

(((TS=(headache*) AND TS=(tension-type)) OR (TS=(headache*) AND TS=(“tension type”)) OR (TS=(idiopathic) AND TS=(headache*)) OR (TS=(stress) AND TS=(headache*)) OR (TS=(tension) AND TS=(headache*)) OR (TS=(pressure) AND TS=(headache*)) OR TS=( “pressure headache”) OR TS=(“pressure headaches”) OR (TS=(psychogenic) AND TS=(headache*)) OR (TS=(tension-vascular) AND TS=(headache*)) OR (TS=(“tension vascular”) AND TS=(headache*)) OR TS=(“tension vascular headache”) OR TS=(“tension vascular headaches”) OR (TS=(post-traumatic) AND TS=(headache*)) OR (TS=(“post traumatic”) AND TS=(headache*)) OR (TS=(cervicogenic) AND TS=(headache*)) OR (TS=(migraine) AND TS=(disorder*)) OR TS=(migraine*) OR (TS=(migraine) AND TS=(headache*)) OR (TS=( “acute confusional”) AND TS=(migraine*)) OR TS=(“status migrainosus”) OR TS=(hemicrania) OR TS=(“status hemicranicus”) OR TS=(“familial migraine”) OR (TS=(migraine) AND TS=(disorder*)) OR (TS=(hemicrania) AND TS=(migraine*)) OR (TS=(migraine) AND TS=(variant*)) OR (TS=(sick) AND TS=(headache*)) OR (TS=(abdominal) AND TS=(migraine*)) OR TS=(“secondary headache disorders”) OR (TS=(“headache disorders”) AND TS=(secondary)) OR (TS=(“headache disorder”) AND TS=(secondary)) OR TS=(“secondary headache disorder”) OR TS=(“secondary headache”) OR TS=(“secondary headaches”) OR (TS=(cervical) OR TS=(“migraine syndromes”)) OR TS=(“chronic tension headache”)) AND (TS=(“dry needle”) OR TS=(“dry needling”) OR TS=(“dry needles”) OR TS=(dry-needl*) OR TS=(needl*) OR TS=(pharmacopuncture) OR TS=(acupuncture) OR TS=(“intramuscular stimulation”) OR TS=(“intramuscle stimulation”) OR TS=(“intramuscular stimulations”) OR TS=(“intramuscle stimulations”)))

| **Search strategy for Embase^®^** |
| --- |

(((headache*:ti,ab AND tension-type:ti,ab) OR (headache*:ti,ab AND “tension type”:ti,ab) OR (idiopathic:ti,ab AND headache*:ti,ab) OR (stress:ti,ab AND headache*:ti,ab) OR (tension:ti,ab AND headache*:ti,ab) OR (pressure:ti,ab AND headache*:ti,ab) OR “pressure headache”:ti,ab OR “pressure headaches”:ti,ab OR (psychogenic:ti,ab AND headache*:ti,ab) OR (tension-vascular:ti,ab AND headache*:ti,ab) OR (“tension vascular”:ti,ab AND headache*:ti,ab) OR “tension vascular headache”:ti,ab OR “tension vascular headaches”:ti,ab OR (post-traumatic:ti,ab AND headache*:ti,ab) OR (“post traumatic”:ti,ab AND headache*:ti,ab) OR (cervicogenic:ti,ab AND headache*:ti,ab) OR (migraine:ti,ab AND disorder*:ti,ab) OR migraine*:ti,ab OR (migraine:ti,ab AND headache*:ti,ab) OR (“acute confusional”:ti,ab AND migraine*:ti,ab) OR “status migrainosus”:ti,ab OR hemicrania:ti,ab OR “status hemicranicus”:ti,ab OR “familial migraine”:ti,ab OR (migraine:ti,ab AND disorder*:ti,ab) OR (hemicrania:ti,ab AND migraine*:ti,ab) OR (migraine:ti,ab AND variant*:ti,ab) OR (sick:ti,ab AND headache*:ti,ab) OR (abdominal:ti,ab AND migraine*:ti,ab) OR “secondary headache disorders”:ti,ab OR (“headache disorders”:ti,ab AND secondary:ti,ab) OR (“headache disorder”:ti,ab AND secondary:ti,ab) OR “secondary headache disorder”:ti,ab OR “secondary headache”:ti,ab OR “secondary headaches”:ti,ab OR (cervical:ti,ab OR “migraine syndromes”:ti,ab) OR “chronic tension headache”) AND (“dry needle”:ti,ab OR “dry needling”:ti,ab OR “dry needles”:ti,ab OR dry-needl*:ti,ab OR needl*:ti,ab OR pharmacopuncture:ti,ab OR acupuncture:ti,ab OR “intramuscular stimulation”:ti,ab OR “intramuscle stimulation”:ti,ab OR “intramuscular stimulations”:ti,ab OR “intramuscle stimulations”:ti,ab))
